# Supplementary material for: Splice-Junction-Based Mapping of Alternative Isoforms in the Human Proteome
Source: Cell Rep. Author manuscript; Available in PMC 2020 Jan 15. (PMC6961840; doi:10.1016/j.celrep.2019.11.026)
Supplement: 2 [file NIHMS1546469-supplement-2.zip › DF1/PXD010154/Pancreas/Pancreas_5_MINK1_AATAAGAGAAEAAGEGAAAAAGGHAG.pdf]

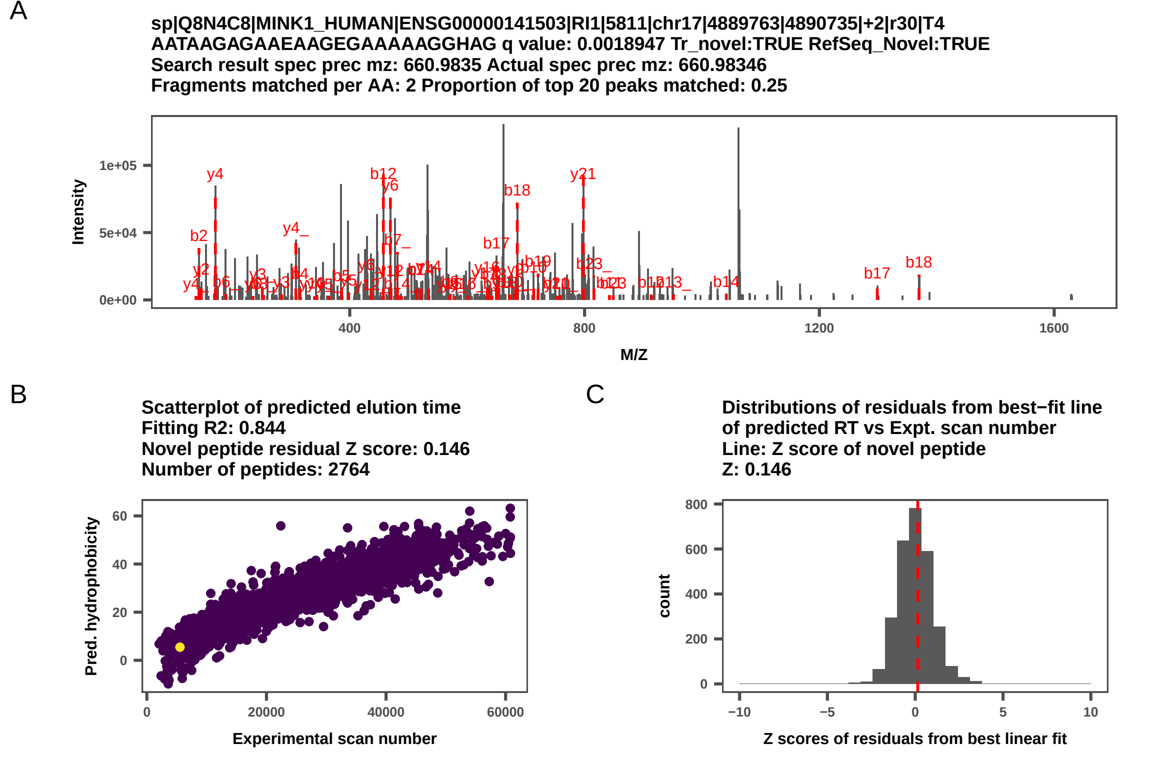
A
sp|Q8N4C8|MINK1_HUMAN|ENSG00000141503|RI1|5811|chr17|4889763|4890735|+2|r30|T4
AATAAGAGAAEAAGEGAAAAAGGHAG q value: 0.0018947 Tr_novel:TRUE RefSeq_Novel:TRUE
Search result spec prec mz: 660.9835 Actual spec prec mz: 660.98346
Fragments matched per AA: 2 Proportion of top 20 peaks matched: 0.25
Intensity
1e+05
5e+04
0e+00
400
800
1200
1600
M/Z
y4
b2
y4_
b12
y6
b7_
b18
b17
y21
b19
b23_
b17
b18
b14
B
Scatterplot of predicted elution time
Fitting R2: 0.844
Novel peptide residual Z score: 0.146
Number of peptides: 2764
Pred. hydrophobicity
60
40
20
0
0
20000
40000
60000
Experimental scan number
C
Distributions of residuals from best−fit line
of predicted RT vs Expt. scan number
Line: Z score of novel peptide
Z: 0.146
count
800
600
400
200
0
−10
−5
0
5
10
Z scores of residuals from best linear fit
